# Description of an alternative method for optimal and comfortable two-handed face mask ventilation: the transverse mandibular technique

**DOI:** 10.1186/s13054-020-02999-z

**Published:** 2020-05-26

**Authors:** Francois Lemay, Jeremy Cooper

**Affiliations:** 1 Département d’anesthésiologie et de soins intensifs, Université Laval, Québec, Canada; 2 Centre Hospitalier Universitaire de Québec, Hôtel-Dieu de Québec, Québec, Canada; 3 Green Lane Department of Cardiothoracic and ORL Anaesthesia, Auckland City Hospital, Auckland, New Zealand

To the Editor,

Recent recommendations stress the importance of avoiding aerosolization while attempting rescue facemask ventilation (FMV) with COVID-19 patients [1]. In addition, major difficult airway algorithms already highlight the importance of oxygenation rather than intubation, and many include best attempts at facemask ventilation (FMV) while progressing in cannot intubate cannot oxygenate situations [2, 3].

Best attempt at FMV may be challenging, and two major techniques have been so far well described for two-handed FMV: the double CE-grip and the thenar eminence techniques, or VE-grip [4]. We would like to share an alternative technique that provides good FMV conditions through improved jaw thrust, mask seal and ergonomic comfort. We would describe this technique as the *transverse mandibular technique* (Fig. 1), for which we have not found any description in the literature. We believe it is already performed by some clinicians and that its use and potential benefits should be discussed.

**Fig. 1:**
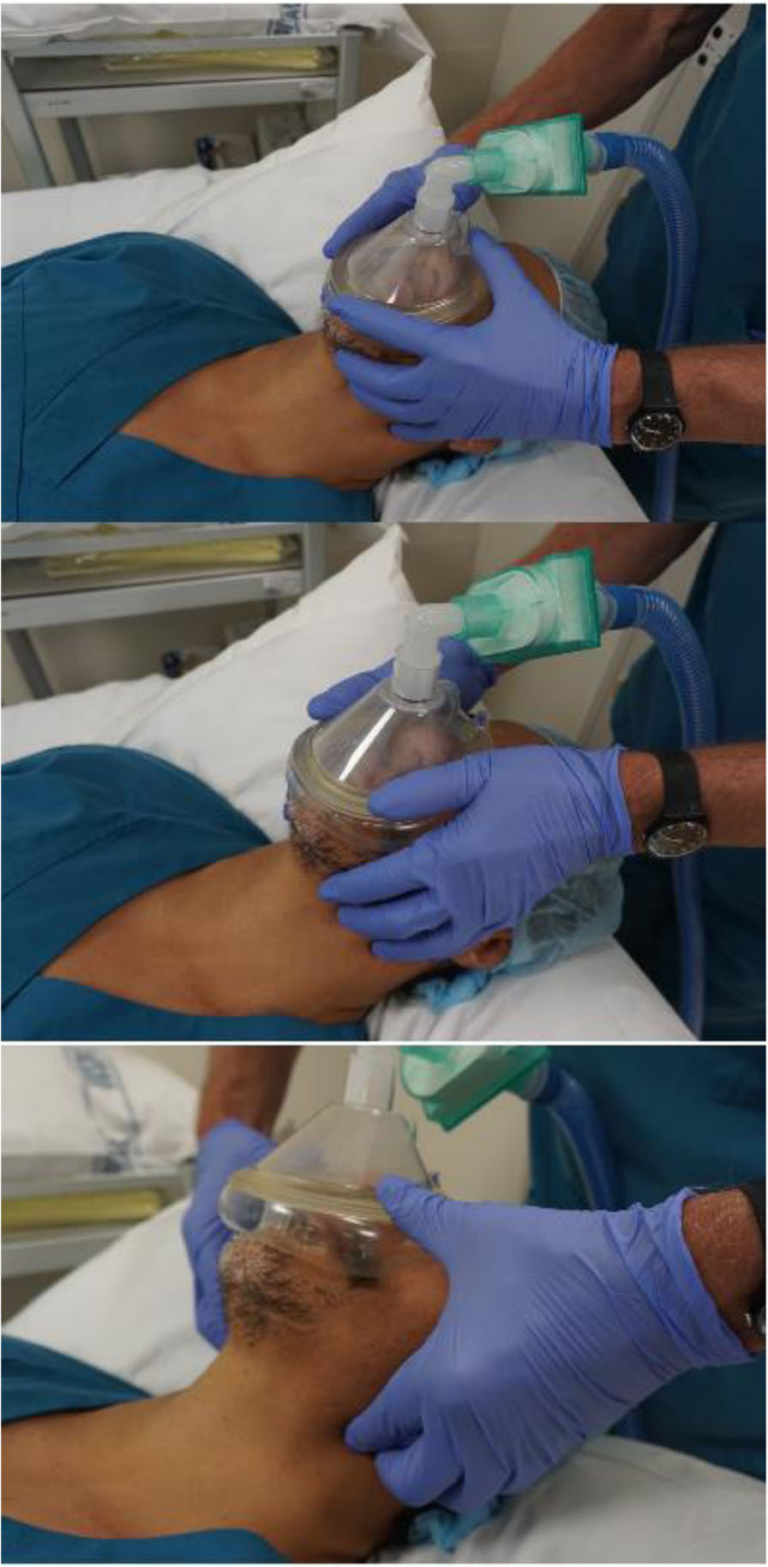
Two-handed positions for face mask ventilation. From top to bottom: double CE-grip, thenar eminence and transverse mandibular techniques

In this technique, emphasis is on mandibular advancement and mask seal. Fingers of both hands (mainly through the index and middle fingers) are placed transversally under the angle of the mandible to achieve an optimal jaw thrust. The thumbs are positioned transversely over the middle of a standard facemask on both sides, pushing down to achieve proper facial seal.

We believe the transverse mandibular technique has some advantages:

1. The strongest fingers in both hands are used to make an appropriate jaw thrust.
2. The thumb grip is potentially less tiring. In other techniques, lateral pressure must be used with the thumbs, which can be difficult especially in smaller hands.
3. The wrists are kept straight over a wide range of table height. This is relevant as the hands have been shown to have a stronger grip with the wrists in a neutral position [5].
4. The position of the hands fits a wide range of facemasks and patients’ sizes and can be used in both adult and paediatric patients.
5. It can be performed standing in front of the patient, which can be useful in critical situations when many practitioners are managing the airway.

It is important that one uses a technique that he or she is comfortable with. Whatever the primary FMV technique, tiring practitioners might want to alternate or combine techniques. We would suggest that practitioners consider the technique advocated in this correspondence as they may find it useful in difficult FMV contexts.

## Data Availability

Not applicable.

## Abbreviation

FMV: Facemask ventilation
